# Risk of Vaginal Infections at Early Gestation in Patients with Diabetic Conditions during Pregnancy: A Retrospective Cohort Study

**DOI:** 10.1371/journal.pone.0155182

**Published:** 2016-05-11

**Authors:** Julian Marschalek, Alex Farr, Herbert Kiss, Michael Hagmann, Christian S Göbl, Marie-Louise Trofaier, Verena Kueronya, Ljubomir Petricevic

**Affiliations:** 1 Department of Obstetrics and Gynaecology, Division of Obstetrics and Fetomaternal Medicine at the Medical University Vienna, Vienna, Austria; 2 Section for Medical Statistics (IMS), Centre of Medical Statistics, Informatics and Intelligent Systems at the Medical University Vienna, Vienna, Austria; Fred Hutchinson Cancer Center, UNITED STATES

## Abstract

Pregnant women with gestational diabetes mellitus (GDM) are reported to be at increased risk for infections of the genital tract. This study aimed to compare the prevalence of asymptomatic bacterial vaginosis (BV) and *Candida* colonization at early gestation between pregnant women with and without diabetic conditions during pregnancy. We included data from 8, 486 singleton pregnancies that underwent an antenatal infection screen-and-treat programme at our department. All women with GDM or pre-existing diabetes were retrospectively assigned to the diabetic group (DIAB), whereas non-diabetic women served as controls (CON). Prevalence for BV and *Candida* colonization was 9% and 14% in the DIAB group, and 9% and 13% in the CON group, respectively (n.s.). No significant difference regarding stillbirth and preterm delivery (PTD), defined as a delivery earlier than 37 + 0 (37 weeks plus 0 days) weeks of gestation was found. We could not find an increased risk of colonization with vaginal pathogens at early gestation in pregnant women with diabetes, compared to non-diabetic women. Large prospective studies are needed to evaluate the long-term risk of colonization with vaginal pathogens during the course of pregnancy in these women.

## Introduction

Bacterial vaginosis (BV) is known to be a crucial factor for preterm delivery (PTD), causing up to 40 percent of premature births [1–3]. In addition, there is increasing evidence supporting a causal role of *Candida* colonization in the multifactorial pathway of PTD, since a benefit of treating women with asymptomatic candidiasis has previously been demonstrated [4, 5]. In view of the available literature on the potentially hazardous pathogens of the vaginal microflora, we introduced an antenatal screen-and-treat program at our department in 2004. This simple public health intervention led to a significant reduction of PTD and late miscarriage rates in the general population of pregnant women [6, 7].

So far, Diabetes mellitus (DM) was thought to predispose women to *Candida* colonization. Results from a large population-based study indicate that women with DM are at an increased risk for infections of the lower genital tract and in particular, those with poorly controlled diabetes seem to be at highest risk for acquiring genital infections [8–11].

As most women in Austria are routinely screened for gestational diabetes mellitus (GDM) at 24 to 28 weeks of gestation, obstetricians notice an increasing GDM prevalence [12, 13]. However, there is little research evaluating GDM as a risk factor for vaginal *Candida* colonization. Nowakowska et al. reported that the risk of vaginal *Candida* colonization in pregnant women is more than four times higher in women with DM compared to non-diabetic women, also postulating an elevated risk in those with GDM [14].

Concerning bacterial vaginosis even less evidence is available for women with GDM. On the one hand, it appears logically consistent that diabetic pregnant women more likely acquire genital infections, because of poor metabolic control, higher body mass index (BMI) and potentially impaired leucocyte function [15, 16]. Moreover, pregnancy itself harbours an immunocompromised state, leading to an increased risk of vaginal *Candida* colonization [17]. On the other hand, there are data of a population-based study that reported no significant association between GDM and BV, which stands in contrast to the explanatory model [18].

In the present study, we aimed to compare the prevalence of asymptomatic bacterial vaginosis and *Candida* colonization at early gestation in pregnant women with and without diabetic conditions during pregnancy. Secondary outcome variables included PTD, defined as a delivery earlier than 37 + 0 (37 weeks plus 0 days) weeks of gestation and infant birth weight. In view of the contradictory literature, the role of diabetes in the multi-factorial mechanism of vaginal infection and PTD should further be evaluated.

## Materials and Methods

### Patients and groups

The study included retrospectively collected data from all women who presented with singleton pregnancies between January 1, 2005, and January 1, 2014, at the Medical University of Vienna, Department of Obstetrics and Gynaecology. Inclusion criteria were registration for a planned delivery at our department between 10 + 0 (10 weeks plus 0 days) and 16 + 0 (16 weeks plus 0 days) weeks of gestation including the antenatal infection screening that was part of our routine pregnancy care [7]. Delivery without undergoing the antenatal infection screening programme (e.g., intervention refused), as well as registration earlier than 10 + 0 or later than 16 + 0 gestational weeks and previous antibiotic treatment (up to 4 weeks prior to the screening) led to exclusion. Women with missing data, incomplete primary or secondary outcome parameters, were also excluded from the analyses.

For the analyses, women were assigned to one of the following groups: the diabetic group (DIAB), which included those with GDM (or DM) or the control group (CON), which included non-diabetic women. Women of the DIAB group were subdivided into (i) those with pre-existing DM (type I or II), (ii) those with GDM and nutritional dietary or (iii) those with GDM and insulin treatment (IGDM). All non-diabetic women had non-pathologic oral glucose tolerance test (oGTT)-results and negative medical history for diabetic diseases.

### Obstetric management

Pregnancy care was equal in both groups with routine consultations and examinations being performed in gynaecological outpatient clinics, following the national welfare pregnancy care programme that is obligatory for all pregnant women in Austria since the year 1973 [19]. In addition to this routine follow-up, pregnant women with diabetes routinely present at our department every two weeks, beginning in the second trimester of their pregnancy. All women with IGDM, GDM or pre-existing DM underwent a multi-disciplinary treatment approach, including supervision by a physician of the Department of Internal Medicine, Medical University of Vienna. In case of any complications (e.g., suboptimal glycaemic control or pregnancy-related problems), consultations were intensified. Most women of the DIAB group underwent induction of labour at 40 + 0 (40 weeks plus 0 days) weeks of gestation.

### Diabetes screening

Since January 1, 2010, women who had not been previously diagnosed with diabetes, routinely undergo a 75-grams two-hour oGTT as part of routine pregnancy welfare care between 24 and 28 gestational weeks. According to the International Association of Diabetes and Pregnancy Study Groups (IADPSG), GDM is diagnosed in case of at least one out of three (fasting, one-hour, two-hours) elevated plasma glucose values [20]. The following glucose levels lead to the diagnosis of GDM: fasting ≥92 mg/dL (5.1 mmol/L), one-hour post-prandial ≥180 mg/dL (10 mmol/L) and/or two-hour post-prandial ≥153 mg/dL (8.5 mmol/L). Only women that were highly suspicious for undiagnosed type II diabetes, as well as those with a prior history of GDM, underwent diabetes screening earlier than 24 gestational weeks. In case of a fasting plasma glucose level ≥126 mg/dL (7.0 mmol/L) and/or a glycated haemoglobin A1c level of ≥6.5% (≥48 mmol/mol), women were diagnosed with DM. A random plasma glucose level ≥200 mg/dL (11.1 mmol/L) was suggestive of overt diabetes. From January 1, 2005 to December 31, 2009, oGTT was exclusively performed in women with a positive family history for DM or clinical suspicion of GDM [21]. Using a 75-grams two-hour oGTT between 24 and 28 gestational weeks, GDM or impaired glucose tolerance in pregnancy was diagnosed in case of at least one out of three (fasting, one-hour, two-hours) elevated plasma glucose values [20]. During this time period, the following glucose levels led to the diagnosis of GDM: fasting ≥95 mg/dL (5.3 mmol/L), one-hour post-prandial ≥180 mg/dL (10 mmol/L) and/or two-hour post-prandial ≥155 mg/dL (8.6 mmol/L).

### Infection screening

According to our routine protocol, vaginal smears were assessed by sterile swabs from the lateral vaginal wall and posterior fornix vaginae; smears were Gram-stained and microscopically analysed by trained and experienced microbiology staff at our department. The classification of the vaginal microflora followed Nugent et al.[22]. The presence of BV, *Candida species* (*spp*.) and *Trichomonas vaginalis* were assessed. In agreement with our study protocol, women with normal or intermediate vaginal microflora on the examined smears did not receive further treatment. Women with evidence of an asymptomatic vaginal infection underwent treatment within 3 to 5 days after diagnosis. Treatment of BV included clindamycin 2% vaginal cream for 6 days in case of a primary infection, oral clindamycin 0.3 g twice daily for 7 days in case of recurrent BV, local clotrimazole 0.1 g for 6 days in case of vaginal candidiasis, and local metronidazole 0.5 g for 7 days in case of trichomoniasis.[23] Recurrent infections with *Candida spp*. and/or *Trichomonas vaginalis* were retreated. Follow-up smears were obtained after 4 to 6 weeks. All women who were treated for BV, as defined by a Nugent score of 7 to 10, were consequently treated with vaginally applied *Lactobacillus spp*. for 6 days, in order to rebuild the physiological vaginal microflora after antibiotic treatment.[24, 25]

### Outcome variables

The rate of asymptomatic vaginal infections in the vaginal smear, defined by the presence of BV, and/or *Candida spp*. (i.e., spors and/or hyphes), and/or *Trichomonas vaginalis*, served as the primary outcome variable. Secondary outcome variables included PTD and infant birth weight. Relevant data were extracted from obstetric databases, patient charts, and microbiology reports. PTD was defined as a delivery earlier than 37 + 0 (37 weeks plus 0 days) weeks of gestation, due to spontaneous preterm premature rupture of membranes (pPROM), preterm labour and/or iatrogenic PTD through early caesarean section or induction of labour. Late miscarriage was defined as the PTD of an infant with a birth weight of less than 500 g, born in the second trimester. Stillbirth was defined as the term or PTD of an infant that had died in utero and was born with an Apgar score of 0/0/0.

## Data Analysis

The Fisher exact tests were used to compare categorical data. For comparisons of continuous data, Welch’s *t*-test was used. Continuous data are given as Mean (± SD, Standard deviation), unless otherwise stated. Discrete data are presented as Numbers (n) and Percentages (%). A two-sided *p*-value < 0.05 was considered statistically significant without adjustment for multiplicity.

In order to check if the GDM screening regimes change had an impact on the results of our analysis, we conducted the analysis (A) for all available patients, and (B) only for patients who underwent infection screening after January 1, 2010. As the estimated statistics displayed only negligible differences, we herewith report results for all available patients. We also conducted a subgroup analysis, to assess, if the vaginal smear outcomes were associated with certain conditions. This evaluation was done by formulating binary logistic regression models for each outcome with a grouping variable coding for the disjoint groups (GDM, IGDM, IDDM and no diabetic condition) as independent variable. Finally, linear hypotheses specified by the presented contrasts were tested.

All statistical calculations were performed using R Project for Statistical Computing, version 3.1.3 (R Development Core Team, MA, USA). The ethical review board of the Medical University of Vienna approved the study (Amendment to Protocol Number 1101/2014), which was performed in accordance with the Declaration of Helsinki and the guidelines of Good Scientific Practice, as supported by the Head of the Institute. As this study comprises retrospectively collected and analysed data, the ethical review board approved the waiver of informed consent.

## Results

### Patients

Between 2005 and 2014, a total of 20,052 women with singleton pregnancies delivered at our department. Of these, the data of 8,486 women (42.3%) met our inclusion criteria, followed by assignment to the following groups: 1,253 women (14.8%) in the diabetic group and 7,233 women (85.2%) in the control group. Out of the DIAB group, 61.2% of the women were diagnosed with IGDM and 31.7% with GDM with the need for dietary requirements. In addition, 7.1% of the DIAB group had pre-existing diabetes type I or type II.

Mean maternal age of women at the time of delivery was 32.8 (± 5.8) years in the study group and 30.5 (± 6.0) years in the control group. No significant difference with respect to the history of PTD was observed between the groups. The sociodemographic and obstetric patients characteristics are provided in Table 1.

**Table 1:** Characteristics of 8,486 women in the DIAB and CON group.

	DIAB	CON
	N (%) *or* Mean ± SD	N (%)*or* Mean ± SD
**Patients**	1,253 (100)	7,233 (100)
Age at birth (years)*	32.8 (± 5.8)	30.5 (± 6.0)
**Diabetic**^**†**^		
DM Type I	58 (4.6)	-
DM Type II	31 (2.5)	-
GDM	397 (31.7)	-
IGDM	767 (61.2)	-
**Parity**		
Primiparae	374 (29.8)	2872 (39.7)
Multiparae	879 (70.2)	4361 (60.3)
**Tertiary education**^#^	62 (5.0)	658 (9.1)
**Nicotine abuse**	219 (17.5)	1412 (19.5)
**Alcohol abuse**	0 (0)	8 (0.1)
**History of PTD**	2 (0.2)	26 (0.4)
**Mode of delivery**		
Vaginal delivery^Δ^	577 (46.1)	4165 (57.6)
Caesarean section^‡^	618 (49.3)	2685 (37.1)
Vacuum assisted delivery	58 (4.6)	383 (5.3)
**Gestational age at delivery**		
< 23+0	3 (0.2)	55 (0.8)
23+0–27+6	8 (0.6)	67 (0.9)
28+0–31+6	19 (1.5)	68 (0.9)
32+0–36+6	108 (8.6)	492 (6.8)
≥ 37+0	1,115 (89.0)	6,551 (90.6)
**Birth weight**		
< 500 g	0 (0)	41 (0.6)
500–999 g	12 (1.0)	75 (1.0)
1,000–1,499 g	7 (0.6)	68 (0.9)
1,500–2,499 g	80 (6.4)	425 (5.9)
≥ 2,500 g	1,153 (92.0)	6,604 (91.3)

Data are presented as N (%) if not otherwise stated or as

* Mean (± Standard deviation).

^†^ DM Type I—pre-existing diabetes mellitus Type I; DM Type II—pre-existing diabetes mellitus Type II; GDM—gestational diabetes; IGDM—insulin-dependent gestational diabetes

^#^ Tertiary education was defined by academic degree

^Δ^ Including vaginal delivery for breech presentation

^‡^ Including emergency caesarean section

### Vaginal smears

**Fig 1:**
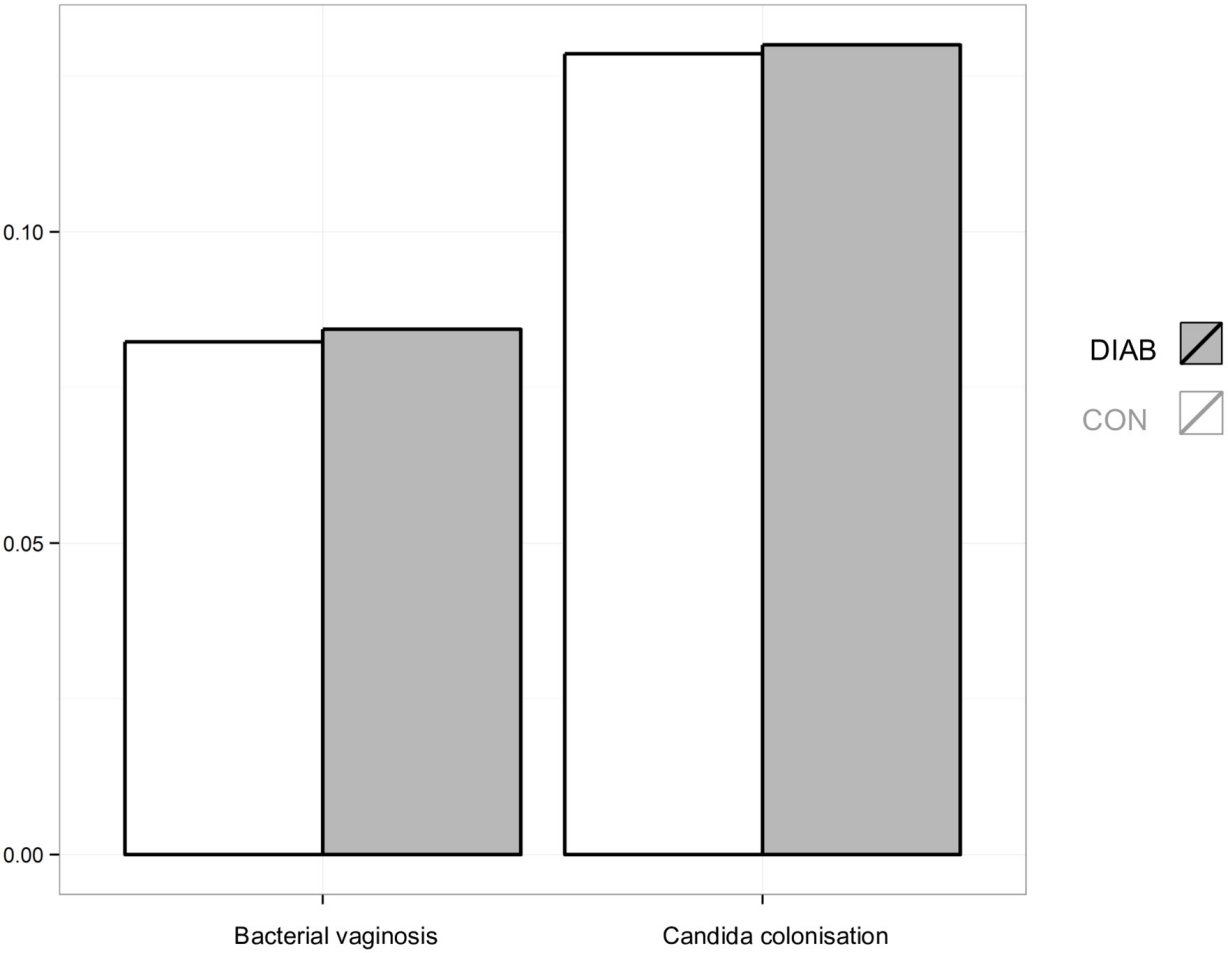
Relative frequency of bacterial vaginosis and *Candida* colonization in 8,486 women of the DIAB and CON group.

Analysis of vaginal screening smears showed no significant difference with respect to normal or abnormal vaginal microflora between the groups (Fig 1). Classification and distribution of vaginal smear results are shown in Table 2. Accordingly, no significant difference was found in the subgroup analysis evaluating the impact of maternal insulin therapy on the vaginal microflora (Table 3).

**Table 2:** Vaginal smear results in 8,486 women of the DIAB and CON group.

	DIAB	CON	OR (95% CI)	p-value
	N (%)	N (%)		
**Vaginal smears**	1,253 (100)	7,233 (100)	-	-
**Normal microflora**	891 (71)	5182 (72)	-	-
**Intermediate microflora**	87 (7)	500 (7)	1.033 (0.832–1.272)	0.750
**Abnormal microflora**				
Bacterial vaginosis (total)	117 (9)	633 (9)	1.074 (0.865–1.324)	0.484
Bacterial vaginosis (alone)	98 (8)	533 (7)		
Bacterial vaginosis + *Candida spp*.	19 (2)	100 (1)		
Bacterial vaginosis + *T*. *vaginalis*	-	-		
*Candida spp*. (total)	171 (14)	975 (13)	1.014 (0.846–1.210)	0.858
*Candida spp*. (alone)	151 (12)	873 (12)		
*Candida spp*. + *T*. *vaginalis*	1 (0)	2 (0)		
*T*. *vaginalis* (total)	7 (0)	45 (1)	-	-
*T*. *vaginalis* (alone)	6 (0)	37 (1)		

OR—odds ratio; CI—confidence Interval

**Table 3:** Subgroup-analysis of 1,253 women in the DIAB group, to assess an association between bacterial vaginosis and *Candida* colonisation and certain diabetes conditions.

	ln(OR)	SE	p-value
**Bacterial vaginosis**			
GDM vs. CON	0.155	0.171	0.654
Pre-existing DM vs. No-insulin^#^	-0.546	0.595	0.646
Insulin* vs. No-insulin^#^	-0.636	0.629	0.584
***Candida* spp.**			
GDM vs. CON	0.031	0.149	0.992
Pre-existing DM vs. No-insulin^#^	0.537	0.327	0.243
Insulin* vs. No-insulin^#^	0.461	0.372	0.460

OR—odds ratio; SE—standard error

* Insulin—including: insulin-dependent gestational diabetes (IGDM), pre-existing diabetes mellitus (DM) Type I and DM Type II (insulin-dependent in pregnancy).

^#^ No-insulin—including: gestational diabetes (GDM), non-diabetic controls (CON)

### Obstetric outcomes

Median gestational age at delivery and median birth weight was 39.0 (IQR 38.0–40.0) weeks and 3,370 (IQR 3,030–3,704) grams in the DIAB group and 39.3 (IQR 38.3–40.4) weeks and 3,290 (IQR 2,940–3,620) grams in the CON group, respectively (All: *p* = 0.0001). No significant difference was found regarding stillbirth (0% versus 0%) and PTD rates (11% versus 9%) when comparing the DIAB group and CON group.

## Discussion

This study aimed to assess the possible association between asymptomatic vulvo-vaginal infection in early pregnancy and hyperglycemic conditions including preexisting diabetes and subsequently diagnosed GDM as well. However, in our large retrospective cohort we were not able to identify an elevated risk for asymptomatic BV or vaginal *Candida* colonization in women affected by diabetes compared to normoglycaemic controls.

Our findings are in contrast to previous observations indicating an increased risk of vaginal *Candida* colonization or infection for women suffering from diabetic disorders [14, 26–28]. In their prospective study Nowakowska et al. assessed the prevalence of *fungi* in 119 diabetic pregnancies and reported a four-fold increased risk of vaginal *Candida* colonization in patients with DM and a two-fold increased risk for women with GDM in comparison with healthy controls. These contradictory results might be explained by the different study designs: In contrast to our study, Nowakowska et al. obtained microbiologic specimens arbitrarily during the course of pregnancy and not at a specific point in time, so that it was not possible to determine the risk for vaginal colonization at early gestation. However, it has to be considered that due to the early assessment of the vaginal microflora in our cohort GDM diagnosis was performed after the time of the infection screening for some subjects. As insulin resistance increases along with gestational age, it might be possible that the susceptibility for vaginal *Candida* colonization in diabetic women rises with duration of pregnancy and poor glycaemic control [26, 27]. In contrast to this hypothesis, previous studies found no association between the mean HbA1c, fasting glucose levels, post-prandial glucose levels and infection rates, when evaluating groups in the same trimester of the pregnancy. No association between the occurrence of vaginal *Candida* colonization and the trimester of pregnancy in diabetic pregnant women has yet been identified as well [14, 28].

In addition, antenatal screening-results in pregnant women with and without need of insulin treatment did not substantially differ from healthy controls in our study. Conversely, Stamler et al. retrospectively compared 65 pregnant women with insulin-dependent diabetes to 65 non-diabetic pregnant controls and supposed insulin dependency as a strong risk factor for infections during pregnancy in general, and vaginal *Candida* colonization in particular [26]. However, underreporting of the control group has to be mentioned as a possible limitation of their study, as they prospectively followed their diabetic cohort, and retrospectively matched their controls.

Actually, there is only sparse data is available assessing the relation between GDM and BV. In a large Danish cohort study, evaluating whether BV was associated with subsequent PTD, low birth weight or perinatal infections, the overall prevalence of BV was 16%, showing a significant association with previous pregnancy termination [18]. Although PTD was also associated with GDM, the authors could not find an association between GDM and BV, comparable to our results. In fact, we were not able to identify any other study investigating GDM as a potential risk factor for BV.

Interestingly, we found no significant differences in obstetric outcomes between the study groups. With the only exception, that non-diabetic women delivered infants at a lower birth weight, born 0.3 weeks later as compared to diabetic women, which could be explained by our pro-active clinical management of diabetic pregnancies with an induction of labour at 40 + 0 gestational weeks. In addition, it is well reported that infants of diabetic pregnant women are born at a higher birth weight [29]. Regarding stillbirth and PTD, we were unable to assess a significant difference with respect to their prevalence in our cohort. This could be a result of the elaborate follow-up programme put in place for women with diabetes, who are advised at obstetric examinations on a regular basis during the second and third trimester of pregnancy.

Some limitations of our study have to be discussed: One of these limitations is caused by the retrospective study design, e.g. missing pre-pregnancy BMI and gestational weight gain. Another is caused by the fact that GDM criteria were different before and after January 2010. In order to assess this potential bias, we decided to perform a sensitivity-analysis of all cases to ascertain data-homogeneity. Conclusions from this analysis suggest that this regime change between the time periods of 2005–2009 and 2010–2014 had no significant impact on the current results of our study. Another limitation of our study is the lack of mean week glucose and glycated haemoglobin A1c levels in diabetic patients, in order to examine an association between glycaemia and prevalence of vaginal infection. A crucial strength of our study is that we analysed a large number of women and that we routinely performed an antenatal infection screening during an asymptomatic state at early gestation. To the best of our knowledge, this study comprises the largest collective of diabetic pregnant women screened for vaginal infection in the literature.

## Conclusion

In conclusion, we could not find an increased risk of colonization with vaginal pathogens at early gestation in pregnant women with diabetes, compared to non-diabetic women. Large prospective studies are needed to evaluate the long-term risk of colonization with vaginal pathogens during the course of pregnancy on the one hand, and the influence of glycaemic control on vaginal infections in women with diabetes on the other.

## Supporting Information

S1 Dataset (XLS)
